# A 15-Year Observational Cohort of Acute Empyema at a Single-Center in Japan

**DOI:** 10.3390/antibiotics13121205

**Published:** 2024-12-11

**Authors:** Nobuhiro Asai, Wataru Ohashi, Yuichi Shibata, Daisuke Sakanashi, Hideo Kato, Mao Hagihara, Hiroyuki Suematsu, Hiroshige Mikamo

**Affiliations:** 1 Department of Clinical Infectious Diseases, Aichi Medical University Hospital, Nagakute 480-1195, Aichi, Japan; 2 Department of Infection Control and Prevention, Aichi Medical University Hospital, Nagakute 480-1195, Aichi, Japan; 3 Department of Pathology, University of Michigan, Ann Arbor, MI 48109, USA; 4 Division of Biostatistics, Clinical Research Center, Aichi Medical University, Nagakute 480-1195, Aichi, Japan; 5 Department of Pharmacy, Mie University Hospital, Tsu 514-0001, Mie, Japan; 6 Department of Clinical Pharmaceutics, Division of Clinical Medical Science, Mie University Graduate School of Medicine, Tsu 514-0001, Mie, Japan; 7 Department of Molecular Epidemiology and Biomedical Sciences, Aichi Medical University, Nagakute 480-1195, Aichi, Japan

**Keywords:** empyema, pleural infection, effusion, potential drug-resistant pathogen, dysbiosis

## Abstract

**Introduction:** Despite the advancements in diagnostic methods and antibiotic treatment, empyema is a critical respiratory infection, showing a high mortality rate of 10–25%. **Patients and Methods:** To evaluate the bacterial etiology and prognostic factors of acute empyema, we conducted this long-term retrospective cohort study at our institute between 2008 and 2022. **Results:** A total of 80 patients were enrolled in this cohort. The median age was 72 years (range 19 to 93 years), and 61 (76%) were male. The most common underlying disease was malignancy, seen in 28 (35%). The mean Charlson comorbidity index (CCI) was 2.7 (±2.4). The 30-day and in-hospital mortality were 9 (11%) and 15 (19%), respectively. Univariate analysis revealed that healthcare-associated infection, inappropriate treatment, high CCI score, performance status (PS) of 2–4, and isolation of potentially drug-resistant (PDR) pathogens were poor prognostic factors. Finally, multivariate analysis showed that high CCI score (*p* = 0.009) and isolation of PDR pathogens (*p* = 0.011) were independent poor prognostic factors for in-hospital death in acute empyema. **Conclusions:** We found that higher CCI scores and isolation of PDR pathogens were independent poor prognostic factors for in-hospital mortality among empyema patients.

## 1. Introduction

Parapneumonic effusion can develop into empyema, which is a severe respiratory infection with a high mortality rate. Pleural effusion is commonly complicated in 0.32 percent of total pneumonia patients [1,2]. While “Hippocrates described the first pleural infection in 500 B.C” [3], empyema remains a critical respiratory infection showing a high mortality rate of 10–25% [3,4,5,6]. Previously, it has been shown that common causative pathogens of empyema were *Mycobacterium tuberculosis and Klebsiella pneumonia* [7]. The advance of an aging society and the emergence of drug-resistant pathogens can influence bacteriology in empyema. Furthermore, the development of newly appeared therapeutic agents such as immunosuppressive agents, biologics, immunotherapy molecular agents for collagen vascular disease, autoimmune diseases, and malignancies might have impacted the altered etiology and bacteriology of empyema. There was no evidence-based guideline in the treatment of empyema due to the lack of a large cohort of empyema, as we previously described [3,8]. This might be one of the reasons that clinicians struggle with empyema patients. We conducted a long-term observational cohort at a single center in Japan to clarify the bacteriology and poor prognostic factors of empyema, which is the first, as far as we know.

## 2. Results

A total of 80 patients were enrolled in this cohort. Patients’ characteristics and clinical outcomes are shown in Table 1. Supplemental Table S1 shows the results of laboratory findings and microbial results between the survival and death groups. The median age was 72 years (range 19 to 93 years), and 61 (76%) were male. In terms of the site of empyema, unilaterality and bilaterality were found in 70 (88%) and 10 (12%), respectively. The mode of onset of empyema was 33 community-acquired (41%) and 47 healthcare-associated infections (59%), respectively.

Regarding the disease severity, median SIRS, qSOFA, SOFA, and APACHE II scores were 2 (range 0–4), 1 (0–3), 2 (0–12), and 11 (2–25), respectively. The most common underlying disease was malignancy, seen in 28 (35%), followed by chronic pulmonary disease in 23 (29%). The mean CCI was 2.7 (±2.4). As for an initial antibiotic agent used, carbapenems were most frequently seen in 29 (36%), followed by penicillin in 25 (31%). While antibiotic combination therapy was initially performed in 23 (29%), monotherapy alone was performed in 57 (71%). Anti-pseudomonal agents were used initially in 60 (75%). Surgical procedures and intrapleural injection of urokinase were performed in 21 (26%) and 14 (18%), respectively. There were no complications regarding the treatments. Regarding the outcomes, the 30-day and in-hospital mortality were 9 (11%) and 15 (19%), respectively. The mean duration of hospitalization and antibiotic treatment was 31 and 33 days, respectively.

### 2.1. Microbial Profiles

Single pathogen and polymicrobial patterns were seen in 61 (76%) and 15 (19%), respectively (Supplemental Table S1). Anaerobic microorganisms were found in 22 (28%). As for the microorganisms isolated in effusion culture, the *Streptococcus anginosus* group (SAG) was most frequently seen in 31 (39%), followed by *Staphylococcus* species in 13 (16%). Regarding the isolated microbial patterns in effusion cultures, single pathogen isolated and polymicrobial patterns were seen in 61 (76%) and 15 (19%), respectively. There was no difference in prognosis between the isolated microbial patterns.

### 2.2. Comparison of Patients’ Profile and Clinical Data Between Survival and In-Hospital Death Groups

We compared patients’ characteristics to clarify the poor prognostic factors for in-hospital death in empyema patients. The survival group tended to be younger than the in-hospital death group. PS and CCI scores were significantly higher in the in-hospital death group than in the survival group. The surgical procedure was more frequently seen in the survival group than in the in-hospital death group.

### 2.3. Analysis of Poor Prognostics Factors for In-Hospital Death in Acute Empyema

Table 2 shows the results of univariate and multivariate analyses. Univariate analysis revealed that healthcare-associated infection, inappropriate treatment, high CCI score, PS of 2–4, and isolation of PDR pathogen were poor prognostic factors. Surgical procedures and anaerobes-associated infection were favorable prognostic factors for in-hospital death among acute empyema patients. Of these, inappropriate treatment, high CCI score, isolation of PDR pathogen, and anaerobes-associated infection were put into multivariate analysis. High CCI scores correlated with PS of 2–4 (*p* < 0.001 by Pearson’s test). Healthcare-associated infection was related to high CCI scores (*p* < 0.001 by Pearson’s test) and PS of 2–4 (*p* < 0.001 by Pearson’s test). Moreover, cases that received surgical procedures showed better PS and lower CCI scores. Thus, to avoid duplicates, healthcare-associated infection, PS of 2–4, and surgical procedures were removed from the further analysis. As a result, high CCI score [Odds ratio (OR) 8.9, 95% confidence interval (CI) 1.7–45.6, *p* = 0.009] and isolation of PDR pathogen (OR 10.1, 95% CI 1.7–59.8, *p* = 0.011) were independent poor prognostic factors for in-hospital death in acute empyema by logistic regression analysis.

### 2.4. Kaplan–Meier Analysis

**Figure 1:**
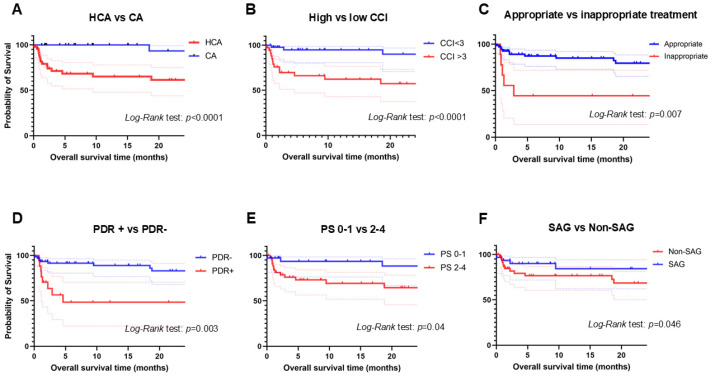
Shows the results of Kaplan–Meier analysis between the two groups. Comparison of overall survival times (OS)s between healthcare-associated and community-acquired empyema (**A**), between high and low Charlson comorbidity index (**B**), between appropriate and inappropriate treatment (**C**), between potential drug-resistant pathogen + and −, (**D**), between performance status 0–1 and 2–4 (**E**), between *Streptococcus anginosus* group (SAG) and non-SAG group (**F**). Dotted lines show 95% confidence interval.

**Figure 2:**
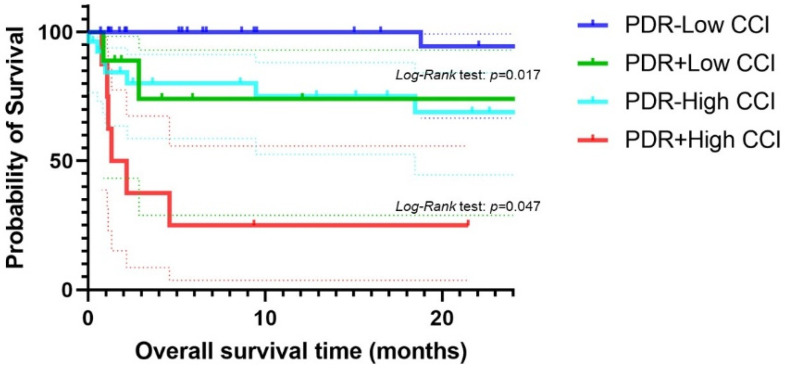
Shows the comparison of OSs among the four groups; patients with high CCI score plus PDR, those with low CCI score plus PDR, those with high CCI score without PDR, and those with low CCI score without PDR. Dotted lines show 95% confidence interval.

Kaplan–Meier analysis displayed OSs in comparison between each group as shown in Figure 1. Patients with healthcare-associated empyema had shorter OSs than those with community-acquired empyema (Figure 1A). Patients with low CCI scores (<3) had longer OSs than those with high CCI scores (≥3) (Figure 1B). Patients who received inappropriate treatment showed shorter OSs than those who received appropriate treatment (Figure 1C). Patients with isolation of PDR pathogens had shorter OSs than those without (Figure 1D). Patients with PS 0–1 showed significantly longer OSs than those with PS 2–4 (Figure 1E). As for the isolated pathogens, patients with *Streptococcus anginosus* group (SAG) displayed significantly longer OSs than those with non-SAG (Figure 1F). There was no significant difference of OSs between patients with or without gram-negative rods, with or without *Staphylococcus* species. Comparing the four groups; patients with high CCI scores plus PDR, those with low CCI scores plus PDR, those with high CCI scores without PDR, and those with low CCI scores without PDR showed significantly shorter OSs than any other groups (Figure 2).

## 3. Discussion

This is the most extended observational cohort study regarding empyema in the world. The mortality rate in our cohort was 19%, similar to previous studies [4,5,6]. The diagnosis for empyema in our cohort was strict. Moreover, patients who were deemed intolerable for surgical intervention were included in the analysis. Thus, the mortality rate was higher than in another cohort, showing 5% of the in-hospital mortality rate [9]. All patients who had surgical treatment survived in our cohort, suggesting our cohort was consistent with the result of the previous study.

As for bacteriology, the most common isolated pathogen from pleural effusion culture was SAG. The patients with SAG exhibited a better outcome than those without. One possible reason is that the origin of SAG-related empyema can be parapneumonic effusion. On the other hand, the origin of *Staphylococcal* and GNR infection-related empyema was bacteremia. Although the mortality rates can differ, there were no patient characteristics differences between the groups. *Enterobacteriaceae* were found in only 4 cases (5%). This result is consistent with the fact that 20% of bacterial origin in empyema is bacteremia [5,6]. The previous report of adults revealed that about 40% of pathogens isolated were *Enterobacteriaceae*, such as *E. coli*, and *Pseudomonas aeruginosa* [7]. This discrepancy in the result can be due to the difference in the population and country in the study. In some studies, regarding pediatric empyema or parapneumonic pleural effusions, common causative pathogens were *Streptococcus pneumoniae* and *S. pyogenes*, accounting for 30–60% of all pathogens in these studies [10,11]. It is well-known that the imbalance of the oral microbiome can cause the development of oral disease as well as cardiovascular disease, pancreatic cancer, inflammatory bowel disease, rheumatic arthritis, and pulmonary diseases [12,13]. Thus, the oral dysbiotic microbiome can lead to the difference in bacteriology in empyema between adults and children [10,11]. Oral care is one of the essential preventive methods for cardiovascular diseases, cancers, as well as respiratory infections.

Nevertheless, broad penicillin and carbapenems were used as an initial antibiotic treatment in 13 (16%) and 29 (36%), respectively, suggesting that overtreatment was performed in some cases. Furthermore, 22 patients (28%) were associated with anaerobic pathogens. We previously documented that most of the anaerobic pathogens from the oral dysbiotic microbiome were susceptible to ceftriaxone, which can be another antibiotic treatment instead of carbapenem or broad-spectrum penicillin for empyema [14]. This overuse of broad-spectrum antibiotics can lead to dysbiosis, which can cause irregular immuno-response in the host, resulting in a poor outcome. Recently, it has been reported that dysbiosis causes severe viral pneumonia in the influenza virus and new coronavirus-19 infection (COVID-19) [15,16]. Clinicians must know that modulating gut dysbiosis can be therapeutic for infectious diseases by using probiotics or avoiding unnecessary broad-spectrum antibiotics. We hypothesized that the isolation of the PDR pathogens could be a sign that dysbiosis occurred in the host. Previous reports demonstrated that dysbiosis in the respiratory and gut microbiomes could induce irregular immune responses, resulting in poor outcomes [17,18,19]. Therefore, patients with the isolation of PDR pathogens had a worse outcome than those without.

We found that a higher CCI score (≥3) was an independent poor prognostic factor for in-hospital death among empyema patients. In terms of a prognostic tool for in-hospital death among empyema, the area under the receiver operating characteristic curve of CCI was 0.761 (95% CI 0.642–0.88, *p* < 0.001), suggesting that CCI can be a predictive tool. The reasons are that higher CCI scores correlate with poorer PSs, and patients with the progressive underlying disease might have gut dysbiosis [16,20], leading to a high mortality rate, even though they had received appropriate treatments.

There were several limitations of our cohort:

1. This is a retrospective study of a small population at a single institute. Therefore, there might have been a selection bias. Also, the sample size might have been small to analyze the prognostic factors, which could have affected the analysis.
2. Hospital-acquired empyema was excluded from the study. It is therefore possible that the population selected in our study could not reflect all empyema patients in our community.
3. We have no data on microbiome analysis even though we mentioned reasons that PDR pathogens can be a poor prognostic factor due to gut dysbiosis. Further study will verify if the hypothesis we thought was correct.
4. We performed pleural effusion culture by microbial testing, not using a polymerase-chain reaction. This might have affected the microbial results in the study.

In conclusion, healthcare-associated empyema demonstrated a poorer prognosis than community-acquired empyema. Logistic regression analysis showed that higher CCI scores and isolation of PDR pathogens were independent poor prognostic factors for in-hospital mortality among empyema patients. Also, empyema patients in the SAG group had significantly longer OSs than those in the non-SAG groups.

## 4. Methods

### 4.1. Study Design and Patients

Our institute is a tertiary teaching hospital with 800 beds, located in the countryside in Aichi prefecture in Japan. The diagnosis of empyema was based on the following criteria as previously described [3,8]. (1) The pleural fluid by thoracentesis was purulent; (2) the pleural effusion culture was positive with elevated white blood cell (WBC) counts showing neutrophil predominance; (3) the presence of clinical symptoms such as fever, cough, sputum, elevated serum WBC counts, and/or CRP. The exclusion criteria are as follows: (1) showed a negative result of pleural effusion culture, and (2) hospital-associated infections. According to previous reports, we evaluated the potential prognostic predictors among acute empyema patients [3,5,6].

### 4.2. Patients’ Conditions and Disease Severity

The Charlson comorbidity index (CCI) evaluates patients’ underlying disease [21]. Eastern Cooperative Oncology Group (ECOG)-Performance Status (PS) assessed patients’ general condition [22]. The disease severity of empyema was scored by *systemic inflammatory reaction syndrome* (SIRS) [23], quick sequential organ failure assessment (qSOFA) [24], sequential organ failure assessment (SOFA) [24], and acute physiology and chronic health evaluation II (APACHE II) scores [25].

### 4.3. Microbial Test and Evaluation

A pleural effusion culture and two sets of blood culture samples were collected from each patient for microbiological examination. The antimicrobial susceptibility of isolated bacterial pathogens was assessed on the basis of the minimum inhibitory concentration according to the Clinical and Laboratory Standards Institute guidelines [26]. Methicillin-resistant *Staphylococcus aureus* (MRSA), *P. aeruginosa*, *Enterobacter*, *Citrobacter*, and extended-spectrum β-lactamase-producing organisms were defined as potentially drug-resistant (PDR) pathogens based on the American Thoracic Society/Infectious Diseases Society of America (ATS/IDSA) guidelines as previously described [14,27,28].

### 4.4. Classification of Community-Acquired or Healthcare-Associated Infection

Community-acquired and healthcare-associated infections were categorized based on the criteria published by ATS/IDSA in 2006, as previously described [27,29].

### 4.5. Definition of Appropriate and Inappropriate Treatment

Antibiotic treatment was classified as appropriate or inappropriate according to whether the identified pathogens were susceptible or resistant, respectively, to the initially prescribed antibiotics as previously described [14,27].

### 4.6. Definition of Medical Condition

Heavy use of alcohol was defined as taking 60 g of alcohol daily for more than five years based on the criteria by the Ministry of Health, Labor and Welfare in Japan as previously described [30].

### 4.7. Statistical Analysis

Descriptive data are reported as means ± standard deviation (SD) or percentages as appropriate. Comparisons between the groups for descriptive summaries were performed using the Mann–Whitney U or Fisher Exact Test as applicable. Kaplan–Meier analysis drew overall survival time (OS), which was calculated from the date of the diagnosis until death from any cause). The *log-Rank* test evaluated the comparison of OSs between the groups. All statistical analyses were carried out using SPSS version 26 for Windows (SPSS Inc., Chicago, IL, USA). Graph Pad Prism v 9.3.1 drew Kaplan–Meier curves. A *p*-value < 0.05 was considered statistically significant. This study was approved by the Institutional Review Board of Aichi Medical University Hospital (2022-123).

## Figures and Tables

**Table 1:** Comparison of patients’ characteristics between the survival and in-hospital death group (n = 80).

Variables	All Patients (n = 80)	Survival Group (n = 65)	In-Hospital Death Group (n = 15)	*p*-Value
Mean age (years ± SD)	69.2 ± 14.3	68.2 ± 15.4	73.7 ± 8.1	0.257 †
Median age (years, range)	72 (19–93)	71 (19–93)	76 (55–85)	-
Male gender (n, %)	61 (76)	49 (75)	12 (80)	0.705
Smoking history (n, %)				
Current smoker	12 (15)	10 (15)	2 (13)	1.000
Ex-smoker	42 (52)	34 (52)	8 (54)	0.943
Never smoker	23 (29)	18 (28)	5 (33)	0.663
Unknown	3 (4)	3 (5)	0	1.000
Location of empyema (n, %)				
Unilateral	70 (88)	59 (91)	11 (73)	0.066
Bilateral	10 (12)	6 (9)	4 (27)	0.066
Single lesion	39 (49)	30 (46)	9 (60)	0.334
Multiple lesions	41 (51)	35 (54)	6 (40)	0.334
Onset of infections (n, %)				
Community-acquired infection	33 (41)	33 (51)	0	<0.001
Healthcare-associated infection	47 (59)	32 (49)	15 (100)	<0.001
Body mass index (kg/m^2^) (mean ± SD)	20.2 ± 5.1	20.4 ± 5.2	19.6 ± 4.9	0.608 †
Performance status scale (mean ± SD)	1.9 ± 1.1	1.6 ± 1.0	3.0 ± 0.7	<0.001
ECOG-PS > 2–4 (n, %)	45 (56)	31 (48)	14 (93)	0.001
Underlying diseases (n, %)				
Heart disease	14 (18)	12 (18)	2 (13)	1.000
Chronic pulmonary disease	23 (29)	16 (25)	7 (47)	0.089
Heavy alcohol consumption	4 (5)	4 (6)	0	1.000
Diabetes mellitus	21 (26)	18 (28)	3 (20)	0.532
Chronic kidney disease	12 (15)	9 (14)	3 (20)	0.688
Hemodialysis	5 (6)	4 (6)	1 (7)	1.000
Gastrointestinal disease	11 (14)	10 (15)	1 (7)	0.68
Collagen vascular disease	6 (8)	6 (9)	0	0.588
Cerebrovascular disease	10 (13)	9 (14)	1 (7)	0.678
Malignancy	28 (35)	16 (25)	12 (80)	<0.001
Paralysis	4 (5)	3 (5)	1 (7)	0.572
Dental diseases	6 (8)	5 (8)	1 (7)	0.892
Charlson comorbidity index (mean ± SD)	2.7 ± 2.4	2.3 ± 2.1	4.5 ± 2.6	0.004 †
Charlson comorbidity index ≥ 3 (n, %)	35 (44)	24 (37)	11 (73)	0.01
Severity of the diseases [median (range)]				
SIRS score	2 (0–4)	2 (0–4)	2 (0–4)	-
Quick SOFA	1 (0–3)	1 (0–3)	1 (0–3)	-
SOFA score	2 (0–12)	2 (0–4)	3 (0–12)	-
APACHE II score	11 (2–25)	10 (2–25)	17 (7–25)	-
Treatment (n, %)				
Surgical intervention	21 (26)	21 (32)	0	0.01
Intrapleural use of urokinase	14 (18)	13 (20)	1 (7)	0.22
Initial antibiotic therapy (n, %)				
Monotherapy	57 (71)	47 (72)	10 (67)	0.754
Penicillin	25 (31)	22 (34)	3 (20)	0.368
Cephems	3 (4)	3 (5)	0	1.000
Carbapenems	29 (36)	22 (34)	7 (47)	0.352
Combination therapy	23 (29)	18 (28)	5 (33)	0.754
Combination therapy with Clindamycin	17 (21)	13 (20)	4 (27)	0.727
Combination therapy with anti-MRSA agents	2 (3)	2 (3)	0	1.000
Others	4 (5)	3 (5)	1 (7)	0.572
Anti-pseudomonal agents use (n, %)	60 (75)	49 (75)	11 (73)	1.000
Duration of				
hospital stay (mean days ± SD)	31.2 ± 17.7	32.6 ± 22.3	33.1 ± 26.9	0.113 †
antibiotics use (mean days ± SD)	32.7 ± 22.9	31.2 ± 17.1	28.7 ± 20.9	0.32 †
Outcome				
Mortality (n, %)				
30-day mortality	9 (11)	-	-	-
In-hospital mortality	15 (19)	-	-	-
Inappropriate treatment (n, %) †	9 (11)	4 (6)	5 (33)	0.003
Isolating PDR pathogens (n, %)	15 (19)	6 (9)	9 (60)	<0.001
Microbial pattern by effusion (n, %)				
Single pathogen isolated	61 (76)	51 (78)	10 (67)	0.332
Polymicrobial pattern	15 (19)	13 (20)	2 (13)	0.724
Mixed with anaerobic and anaerobic pathogens	7 (9)	7 (11)	0	0.337
Anaerobic pathogen isolated	22 (28)	21 (26)	1 (7)	0.056

APACHE II, acute physiology and chronic health evaluation II; ECOG, Eastern Cooperative Oncology Group; MRSA, methicillin-resistant *Staphylococcus aureus*; PDR, potential drug-resistant; PS, performance status; SD, standard deviation; SIRS, systemic inflammatory reaction syndrome; SOFA, sequential organ failure assessment. † The analyses were performed by Mann-Whitney U.

**Table 2:** Poor prognostic factors for in-hospital death among acute empyema patients (n = 80).

Variables	Univariate Analysis			Multivariate Analysis		
	Odds Ratio	95% CI	*p*-Value	Odds Ratio	95% CI	*p*-Value
Inappropriate treatment	7.6	1.7–33.3	0.003	-	-	-
Isolation of PDR pathogen	9.3	2.7–32.6	<0.001	10.1	1.7–59.8	0.011
High CCI score (≥3)	4.7	1.3–16.4	0.01	8.9	1.7–45.6	0.009
Anaerobius associated infection	0.2	0.0–1.2 *	0.056	-	-	-

CCI, Charlson comorbidity index; CI, confidence interval; PDR, potential drug-resistant. * 0.02–1.2.

## Data Availability

The original contributions presented in the study are included in the article, further inquiries can be directed to the corresponding author.

## Supplementary Materials

The following supporting information can be downloaded at: https://www.mdpi.com/article/10.3390/antibiotics13121205/s1, Table S1: Comparison of laboratory findings and microbial results between patients in the survival and in-hospital death group.
