# Supplementary material for: A 15-Year Observational Cohort of Acute Empyema at a Single-Center in Japan
Source: Antibiotics (Basel). 2024 Dec 11;13(12):1205. doi: 10.3390/antibiotics13121205 (PMC11672654; doi:10.3390/antibiotics13121205)
Supplement: Supplementary file 1 [file antibiotics-13-01205-s001.zip › antibiotics-3287391-supplementary.pdf]

Table S1. Comparison of laboratory findings and microbial results between patients in the survival and in-hospital death group (n=80).

Variables	All patients (n=80)	Survival group (n=65)	In-hospital death group (n=15)	*p*-value
Laboratory findings (mean±SD）				
White blood cell counts (/μl)	17427.5±10431.1	18,709±10,491.3	11,873±8,827.0	0.016
Hemoglobin (g/dl)	11.4±2.1	11.5±2.1	10.8±2.2	0.299
Hematocrit (%)	34.6±6.4	34.8±6.4	33.6±6.8	0.55
Platelet ($\times 10^4/\mu$l)	34.6±14.2	36.4±13.7	26.8±14.7	0.031
Sodium (mEq/l)	136.4±4.1	136.0±3.9	138.1±3.9	0.138
Potassium (mEq/l)	4.2±0.6	4.2±0.5	4.3±0.7	0.456
Total bilirubin (mg/dl)	0.9±1.0	0.8±0.5	1.4±2.0	0.257
Blood urea nitrogen	27.0±20.5	25.7±20.7	32.7±20.1	0.243
Creatinine (mg/dl)	1.4±1.9	1.4±2.1	1.4±1.0	0.946
Albumin (g/dL)	2.6±0.7	2.6±0.7	2.5±0.6	0.645
C-reactive protein (mg/dl)	20.4±10.5	20.6±10.3	19.5±12.0	0.751
Pathogen isolated by effusion culture (n,%)				
Streptococcus anginosus group	31 (39)	28 (43)	3 (20)	0.098
Other streptococcus species	11 (14)	9 (15)	2 (13)	1.000
Staphylococcus species	13 (16)	10 (17)	3 (20)	0.702
Enterococcus species	2 (3)	1 (2)	1 (7)	0.342
Fusobacterium species	10 (13)	10 (15)	0	0.104
Prevotella species	3 (4)	3 (5)	0	1.000
Parvimonas species	2 (3)	2 (3)	0	1.000

Escherichia coli	1 (1)	1 (2)	0	1.000
Klebsiella species	1 (1)	1 (2)	0	1.000
Pseudomonas species	2 (3)	1 (2)	1 (7)	0.342
Bacteroides species	2 (3)	2 (3)	0	1.000
Other gram-negative rods	5 (6)	2 (3)	3 (27)	0.043
Others	17 (21)	13 (20)	4 (27)	0.727

SD, standard deviation.
